# Molecular characteristics of bovine norovirus and nebovirus in Swedish dairy herds

**DOI:** 10.1186/s13028-025-00830-9

**Published:** 2025-11-13

**Authors:** Madeleine Tråvén, Anna Svensson, Charlotte Axén, Malin Åberg, Aude Leclerc, Camilla Björkman, Karin Werme, Isabel Blanco-Penedo

**Affiliations:** 1 Department of Clinical Sciences, Swedish University of Agricultural Sciences (SLU), PO box 7054, Uppsala, 75007 Sweden; 2 Department of Animal health and antimicrobial strategies, Swedish Veterinary Agency (SVA), Uppsala, 75189 Sweden; 3 École National Vétérinaire de Toulouse, Toulouse, France; 4 Departamento de Ciencia Animal, Universidad de Lleida, Lleida, Spain; 5 Distriktsveterinärerna, Kristianstad, 29165 Sweden; 6 Present Address: Liljevalchsgatan 22A, 15145 Södertälje, Sweden

**Keywords:** Calves, Neonatal enteritis, Phylogeny, Transmission, Virus genotype

## Abstract

**Background:**

Neonatal enteritis is a major cause of losses in dairy calves and bovine norovirus (BNoV) and nebovirus (NeV) are underdiagnosed contributors to this disease. In this study, we report for the first time molecular characteristics of bovine norovirus (BNoV) and nebovirus (NeV) detected in calves in Swedish dairy herds. 700 samples from preweaned calves with and without diarrhea were analysed.

**Results:**

BNoV was more prevalent (19%) than NeV (4.5%), and among BNoV, the GIII.P2 genotype was more frequently detected than the GIII.P1 genotype. These viruses were detected at similar frequencies in calves with and without diarrhea. The 17 NeV partial polymerase gene sequences all clustered with the Bo/NB/80/ USA prototype strain. Also, the molecular epidemiology of BNoV GIII.P1 in a longitudinal study in one dairy herd is reported.

**Conclusions:**

In this study, we describe for the first time molecular characteristics of BNoV and NeV from Swedish dairy herds. The genotypes detected in Swedish dairy calves were similar to those detected in most of the studies from other countries within and outside Europe. Phylogenetic clustering of Swedish virus strains was detected and discussed in relation to virus transmission.

**Supplementary Information:**

The online version contains supplementary material available at 10.1186/s13028-025-00830-9.

## Background

Bovine norovirus (BNoV) and nebovirus (NeV) are bovine enteric caliciviruses, belonging to separate genera within the family *Caliciviridae* [1]. BNoV is closely related to human NoV, which causes epidemics of gastrointestinal disease worldwide. The bovine strains, however, belong to a separate genogroup, GIII, defined phylogenetically by the sequences of the capsid protein (VP1) gene and the RNA-dependent RNA polymerase (RdRp) gene [2]. Within the bovine GIII, two genotypes have been identified in Europe with the GIII.1 prototype strain Bo/Jena/80/DE detected in Germany in 1980 [3], and the GIII.2 strain Bo/Newbury2/76/UK detected in England in 1976 [4]. A divergent GIII.3 NoV has also been described in sheep in New Zealand [5] and GIII.4 in cattle in China and Uruguay [6, 7]. Recently, the classification of noroviruses has been updated to specify the polymerase type, for example GIII.2 [P2] for Newbury2-like strains [8, 9]. Within NeV three genotypes have been identified, with prototype strains Bo/NB/80/USA from a calf in Nebraska, USA [10],, Bo/Newbury1/76/UK detected in England [11] and Bo/DijonA216/06/FR in France [12]. Both BNoV and NeV have since been detected in a number of countries on all continents [13, 14].

Enteric infections are a major cause of morbidity and mortality in young calves. Both BNoV and NeV have been shown experimentally to cause calf diarrhea and enteric pathology in calves [10, 15]. In a field study, both infections were also associated with diarrhea, but were often detected in coinfection with other pathogens [16]. The precise role of these viruses in calf disease remains to be determined, but information to date suggests that NeV is more virulent than BNoV [10, 16], and within BNoV, the GIII.1 appears to cause more severe disease than the GIII.2 [15, 17]. In a previous study we found a BNoV prevalence of 20% and a NeV prevalence of 5% in Swedish preweaned dairy calves, affecting 48% and 16% of the dairy herds, respectively [13]. However, the molecular characteristics and phylogeny of BNoV and NeV strains circulating in Swedish cattle herds are unknown. The aim of this study was to investigate the molecular characteristics of BNoV and NeV from Swedish preweaned dairy calves and to compare them phylogenetically with published sequences. The overall aim was to increase knowledge of viral transmission patterns in order to prevent disease.

## Methods

### Sampling

Fecal samples were collected rectally from preweaned dairy calves in four field studies designed for other purposes [18–21]. In three studies, 97 herds were visited once and five calves were randomly selected for sampling [18–20]. In the fourth study, 17 calves were sampled weekly until weaning for within-herd epidemiology over time [21]. Samples were transported to the laboratory on ice the same day and kept refrigerated until aliquoting. In addition, pooled fecal samples from outbreaks of calf diarrhea (*n* = 50) were analyzed. These samples were sent by post to the diagnostic laboratory at the Swedish Veterinary Agency (SVA). 1–4 preweaned calves with diarrhea were sampled per herd and pooled by herd at the laboratory. In total, 700 samples were collected from different parts of Sweden during a 7-year period from 2005 to 2012. Fecal suspensions 1:10 in sterile physiologic saline were prepared and stored at −20 °C until analysis.

### RT-PCR for BNoV and NeV

Samples were thoroughly mixed and centrifuged for 10 min at 2000 RPM to spin down coarse materials. RNA was extracted from 140 µL of supernatant using QIAamp viral RNA Mini kit (Qiagen) according to the manufacturer’s protocol. One-step RT-PCRs directed to the RNA-dependent RNA polymerase (RdRp) were applied for both viruses. RT-PCR for NeV was performed as previously described [22], using primers NBU-F/R returning a 549 bp amplicon. RT-PCR for BNoV was done using forward primer J11U [23] and reverse primer CBECU-R [22], yielding a 609 bp product. The RT was performed with AMV reverse transcriptase (Promega) at 43 °C for 60 min and terminated by 94 °C for 3.5 min. The PCR was performed with GoTaq Flexi DNA polymerase (Promega) as 35–38 cycles of denaturation at 94 °C for 40 s, annealing at 53 °C for 50 s, extension at 72 °C for 1 min and final extension at 72 °C for 10 min. PCR products were visualized on 2% agarose gels.

RT-PCR amplification of the capsid gene of BNoV was done with primers MNoroF (5’ GCGACACCCTTCCCGATT 3’) and MNoroR (5’ AGAGCGAGCCTGCAACTCC 3’). RT was performed as above, PCR cycling conditions were 94 °C for 40 s, 50 °C for 1 min, 72 °C for 2 min for 35 cycles and final extension at 72 °C for 10 min, with an expected product of 1705 nt.

### Sequencing and phylogenetic analyses

PCR products were purified from the gel and sequenced in both directions (Macrogen Europe, The Netherlands). Partial RdRp sequences were edited and aligned using BioEdit 7.2 [24] and MEGA 11 [25]. Phylogenetic trees were constructed using Neighbor-joining method with default settings in MEGA 11 and bootstrap values calculated for 1000 replicates. Reference sequences were obtained at https://blast.ncbi.nlm.nih.gov/.

## Results

### Bovine norovirus

The prevalence of BNoV was 19% (93/485) in three of the studies combined [18–20]. BNoV-positive herds were located in southern, central and the southern parts of northern Sweden. Information on diarrhea at the time of sampling was available in two of the studies [18, 20]. Diarrhea was present in 26% of BNoV-positive and 27% of test negative calves. In the longitudinal study conducted in one herd [21], BNoV was detected in 28% (46/165) of the samples. All BNoV sequences from this herd were of the GIII.P1 genotype. In this herd, 13 out of 17 calves tested BNoV positive with the sampling strategy once per week. Among these 13 calves, three were positive on two consecutive weeks, all at age 1–15 days, whereas two calves tested positive again late in the milk-feeding period (age 37–69 days). Eight calves tested positive only once. In the samples sent to SVA from outbreaks of calf diarrhea, BNoV was detected in 14% (7/50) of the pools.

**Fig. 1:**
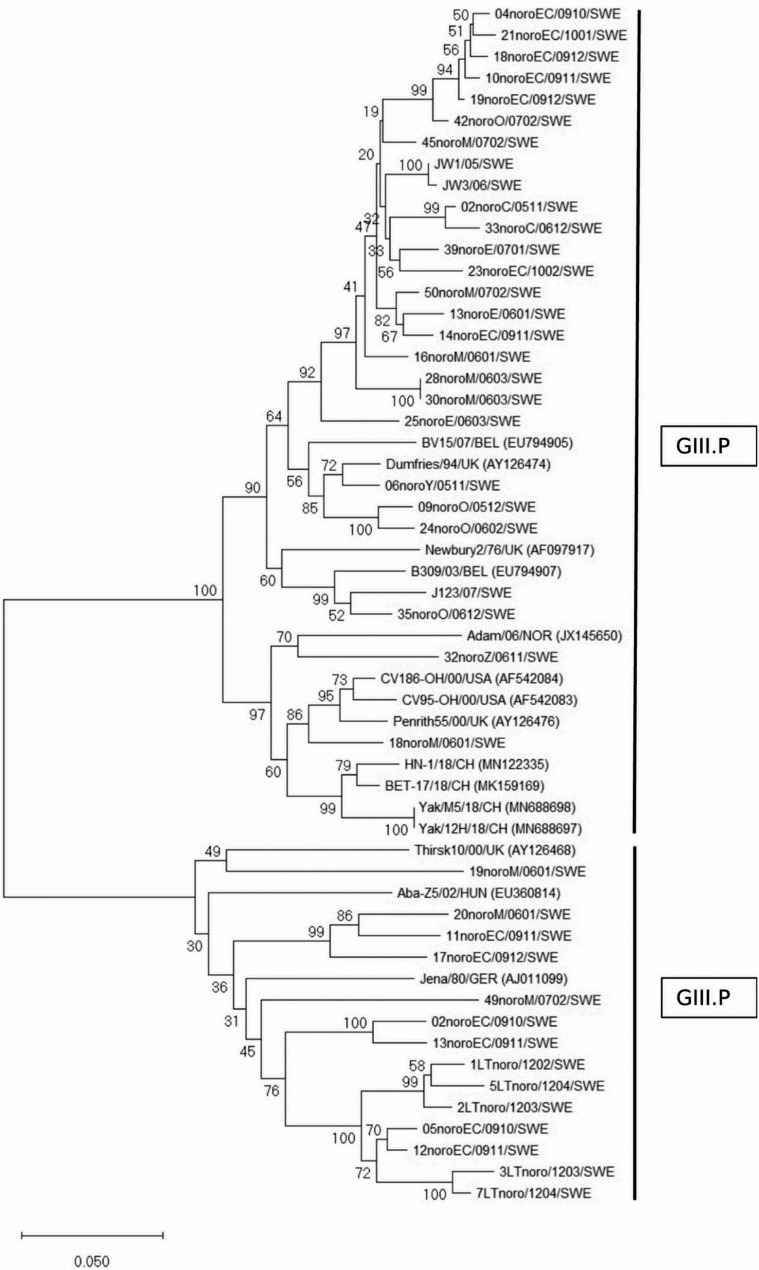
Bovine norovirus RdRp. Neighbor-joining phylogenetic tree of a 563 bp nt section of the RNA-dependent RNA polymerase gene of Swedish bovine norovirus and reference strains. Bootstrap values of nodes based on 1000 replicates, values > 70% shown. Analyses were performed in MEGA 11. Sequences designated SWE were detected in this study. Strain designations represent geographic region (C, E, M, O, Y, Z, EC, Additional file 1), year (06, 07 etc.) and year month (0912, 1203 etc.) of sampling. Bar represents nt substitutions per site

In this study, partial RdRp sequences were detected for 41 BNoV (563 nt, Fig. 1). Fourteen of the Swedish BNoV sequences belonged to genogroup GIII.P1 (Jena-like) and 27 to genogroup GIII.P2 (Newbury2-like). The Swedish GIII.P2 strains clustered together with 88.1–100% nt identity, except for two strains that formed a separate cluster together with reference sequences from Norway, USA and China (87.4–89.9% nt identity with the majority). Here the term ‘reference’ is used in a broader sense to refer to previously published sequences. The Swedish GIII.P1 strains were more diverse with 73.5–98.8% nt identity. The Swedish GIII.P1 cluster showed 73.7–77.8% nt identity with the Swedish GIII.P2 strains. A tendency for geographic clustering was seen for some Swedish strains with strain designations M, C, E, O, EC (Fig. 1). For geographic location of the regions (counties), see Additional file 1. Concerning the sequences designated EC, the majority of the samples were from regions E and C with a few from regions D and AB (Additional file 1), Information on the location of individual herds, however, was not available. For the samples from diarrhea outbreaks no information was provided about geographic location of the herds, thus the sequences from this material lack geographical designation. The herds supplying samples JW and LT were located in regions N and C, respectively. Among the GIII.P2 strains, two samples were from sampling occasions four months apart in the same herd (JW1/05, JW3/06, 99.6% nt identity). Among the GIII.P1 strains, 5 were from different dates in the same herd sampled up to 2 months apart (1LT/1202, 2LT/1203, 3LT/1203, 5LT/1204, 7LT/1204). These 5 strains belonged to two variants with 91.1–93.1% nt identity between variants (Fig. 1). Within variant, nt identity was 97.8% (LT3, 7) and 96.9–97.5% (LT1, 2, 5).

**Fig. 2:**
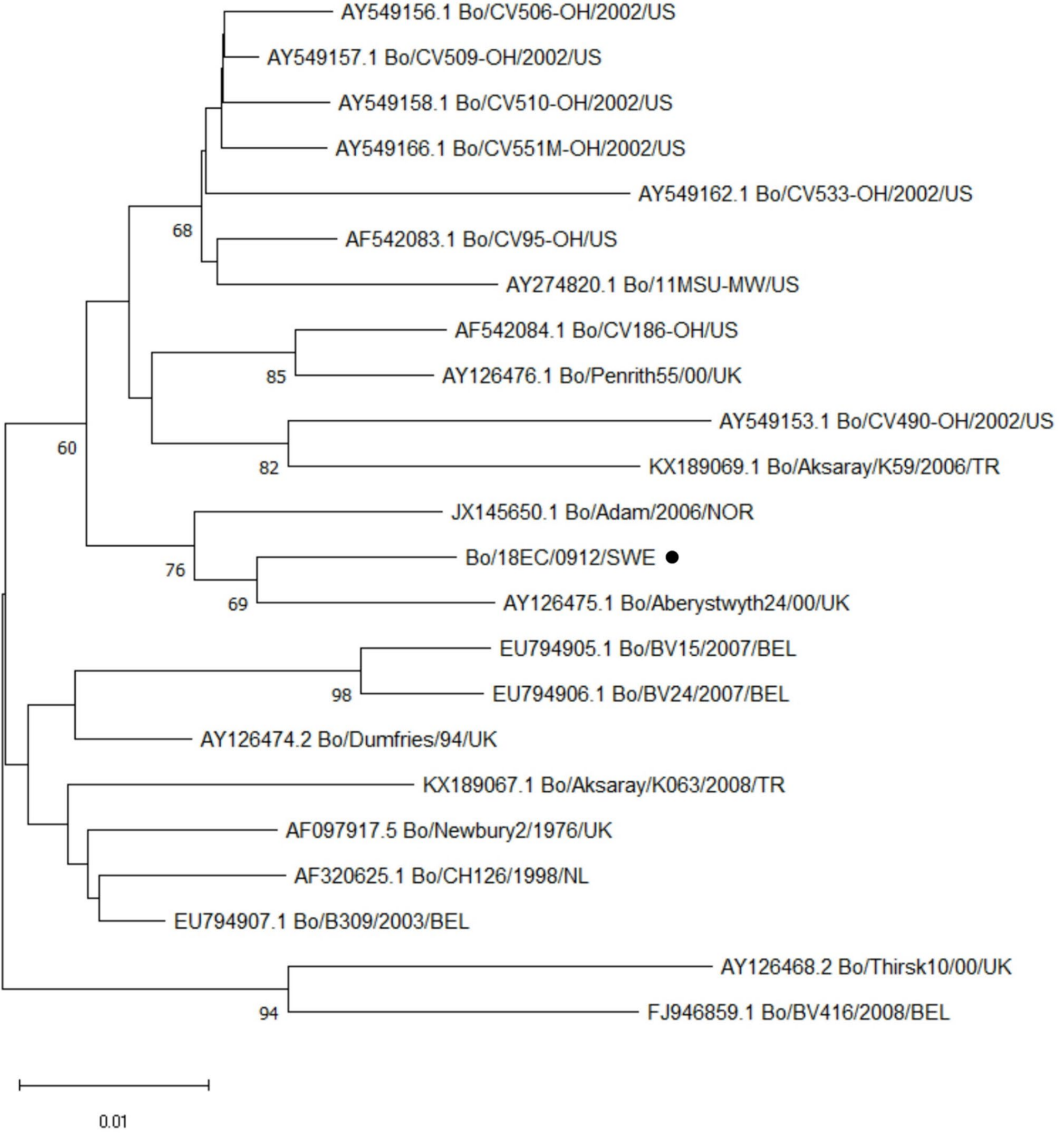
Bovine norovirus VP1. Neighbor-joining phylogenetic trees of the 522 aa complete VP1 (major capsid protein) sequence of a Swedish bovine norovirus and reference strains. Bootstrap values of nodes based on 1000 replicates, values >60% shown. Analyses were performed in MEGA 11, GIII.2 sequences shown. The sequence designated SWE was detected in this study. Bar represents aa substitutions per site

The complete VP1 (major capsid protein) gene was sequenced from a calf sampled in 2009 (Bo/18EC/0912/SWE). This 1569 nt sequence shared 81.8–89.2% nt identity (94.1–97.7% aa identity) with reference GIII.2 VP1 gene sequences and was most closely related to BNoV strains from UK and Norway obtained in 2000 and 2006, respectively (Fig. 2). The Swedish VP1 putative aa sequence shared only 21.2–21.7% identity with GIII.1 VP1 sequences available in GenBank (Additional file 2), and 46.5% identity with Chinese VP1 sequences representing a divergent VP1 genotype [6].

### Nebovirus

**Fig. 3:**
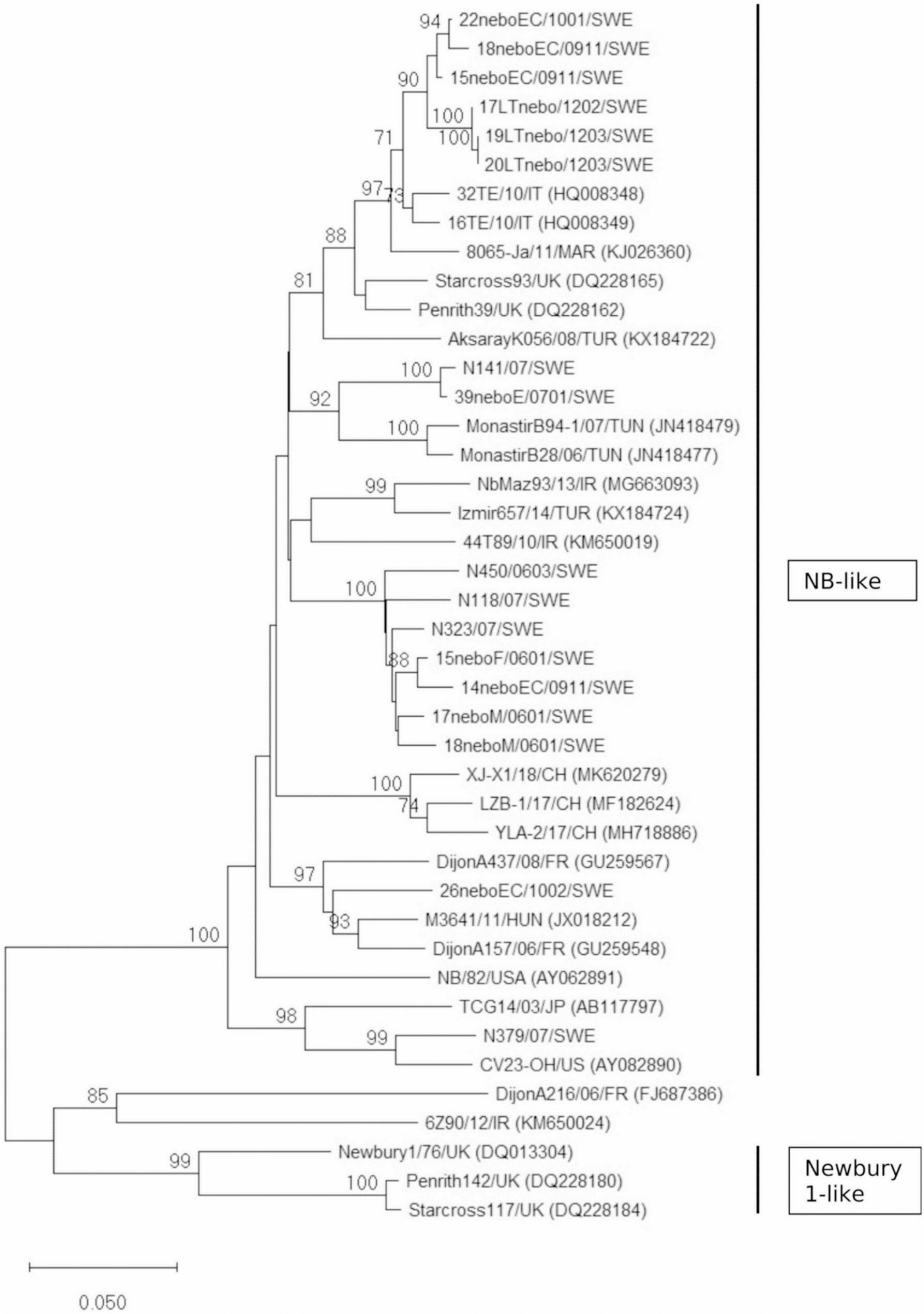
Nebovirus RdRp. Neighbor-joining phylogenetic tree of a 503 bp nt section of the RNA-dependent RNA polymerase gene of Swedish nebovirus and reference strains. Bootstrap values of nodes based on 1000 replicates, values > 70% shown. Analyses were performed in MEGA 11. Sequences designated SWE were detected in this study. Strain designations represent geographic region (E, F, M, EC, Additional file 1), year (07) and year month (0911, 1203 etc.) of sampling. Bar represents nt substitutions per site

The prevalence of NeV in the three studies combined was 4.5% (22/485). In the longitudinal study 3.3% (4/121) NeV was detected, while 10% of the calf diarrhea outbreak pools were positive for NeV. NeV-positive herds were located in southern and central Sweden. Diarrhea was present in 27% of NeV-positive and 26% of negative calves, not significantly different. Partial RdRp sequences were detected for 17 NeV (503 nt, Fig. 3). The Swedish NeV strains showed 81.1–99.2% nt identity, except for 3 strains (17LT/12022, 19LT/1203, 20LT/1203) derived from sampling occasions 1–2 months apart on the same farm, which showed 99.8–100% nt identity. In this herd, only 4 calves out of 17 were positive for NeV at one occasion each (4/121, 3.3%), at age 11–18 days. All Swedish strains clustered with the Bo/NB/80/USA prototype strain, showing 81.8–96.1% nt identity within this genogroup. The Swedish strains were more distantly related to the Newbury1 and DijonA216 genogroups, with 68.5–75.2% and 66.6–72.0% nt identity, respectively. A tendency for geographic clustering was seen for some Swedish strains with strain designations M, EC (Fig. 3).

## Discussion

Here we describe for the first time the molecular characteristics of BNoV and NeV from Swedish dairy herds. BNoV was detected more frequently than NeV, and the prevalences were similar to the findings in our previous study [13]. The higher prevalence of BNoV is also consistent with most studies from other countries where both infections have been investigated: USA [16, 22], France [12], Tunisia [26], Iran [27] and Turkey [28]. Another study from Turkey, however, showed a higher prevalence of NeV [29] and studies from South Korea showed equal prevalence of both infections [30, 31].

BNoV GIII.P2 strains were detected more frequently than GIII.P1, a finding consistent with most of the studies based on partial or complete sequences of RdRp and/or VP1 from other countries: UK [32], The Netherlands [33], USA [22, 34], South Korea [27, 35], Belgium [36, 37], Norway [38], Turkey [28, 29, 39], Argentina [40], China [41] and Uruguay [7].

In this study we report the circulation of BNoV GIII.P1 over a two-month period in a 140-cow dairy herd. The incidence of calf diarrhea in this herd was low. The heterogeneity of the partial RdRp sequences suggests the co-circulation of several GIII.P1 strains. The age, up to two weeks, of calves shedding BNoV was similar to that reported in other studies [7, 12, 13, 16, 35]. Some studies, however, have reported a much higher mean age of shedders [26, 38], possibly due to differences in herd management or sampling strategy. Notably, some calves in our study were positive as early as 1–3 days of age. Repeated shedding later in the milk-feeding period most likely represents reinfections, although longterm shedding of NoV has been reported in immunocompromised humans [42].

Recombination in the BNoV genome near the RdRp/Vp1 junction (GIII.1/GIII.2) is common and such strains have been reported in several studies [25, 40, 43–45, 48]. In this study, recombinant strains were not detected, both the RdRp and VP1 sequence of the strain 18EC/0912/SWE clustered with the Bo/Newbury2/76/UK prototype strain (GIII.2[P2] genotype). Other VP1 sequences were not detected despite the use of several RT-PCRs previously described [41, 43, 45] and other primers designed in this study.

All Swedish NeV strains clustered with the Bo/NB/80/USA prototype strain. This is in agreement with all the studies mentioned above. In addition, partial RdRp sequences of NeV strains from China [46, 47] also clustered with this genotype, while NeV from Brazil and Iran were among the few strains outside the UK and France to cluster with the Bo/Newbury1/76/UK prototype strain [11, 12, 27, 48]. The finding that only 4/121 samples were positive for NeV in the longitudinal study was surprising, given the frequent BNoV infections detected in this herd. This might indicate a shorter period of NeV shedding than for BNoV. Experimental data on NeV shedding beyond the first few days post infection has, to our knowledge, not been reported [10, 15].

BNoV and NeV were detected in herds in different parts of southern and central Sweden and appear to be endemic in these areas. This is consistent with reports from many other countries in Europe and elsewhere. Also, two herds in northern Sweden tested positive for BNoV, although few herds from northern Sweden were included in the study. Partial RdRp sequences are relatively easy to obtain using RT-PCR [22] and therefore abundant in GenBank. The RdRp gene, however, is not optimal for transmission studies since this gene is more conserved for functional reasons, whereas the VP1 gene has a higher variability [37, 48]. We believe, however, that the resolution within the RdRp gene allows some conclusions on transmission of both BNoV and NeV on an overall scale, since sequences from the same country obtained at about the same time tend to cluster. Further, among the Swedish strains we detected clustering by region (strain designations M, C, E, O, EC) and sampling time, suggesting that a large part of the between-herd transmission of the virus occurs locally within regions. Strains from the same herd also tended to cluster with high nt identity within variant (strain designations JW, LT), suggesting within-herd circulation of the virus rather than new introductions in these cases. Some Swedish strains, however, cluster more closely with sequences from the UK, Belgium and USA (BNoV), Italy and USA (NeV).

The Swedish VP1 sequence was most closely related to sequences from the UK and Norway, although the difference in sampling time was quite large. Live cattle imports to Sweden are very limited, but indirect contacts could potentially transmit the infection as caliciviruses are quite stable in the environment [42]. Further analysis of VP1 sequences from Swedish cattle is warranted to clarify transmission patterns between herds and regions. Increased knowledge of calf diarrhea pathogens is important for efficient prevention of these infections.

## Conclusions

BNoV were more prevalent than NeV in Swedish dairy calves and among BNoV, the GIII.P2 genotype was more prevalent than the GIII.P1. Swedish strains showed clustering by region and sampling time. More VP1 sequences, however, are needed to investigate between-herd transmission patterns.

## Supplementary Information

Supplementary Material 1

Supplementary Material 2

## Data Availability

Sequences from this study were deposited in GenBank with accession nos. PP957418, PP957658-PP957684, PP957696-PP957709, PP965309-PP965325.
