# Supplementary material for: Molecular characteristics of bovine norovirus and nebovirus in Swedish dairy herds
Source: Acta Vet Scand. 2025 Nov 13;67:46. doi: 10.1186/s13028-025-00830-9 (PMC12616915; doi:10.1186/s13028-025-00830-9)
Supplement: Supplementary file 1 — Supplementary Material 1 [file 13028_2025_830_MOESM1_ESM.docx]

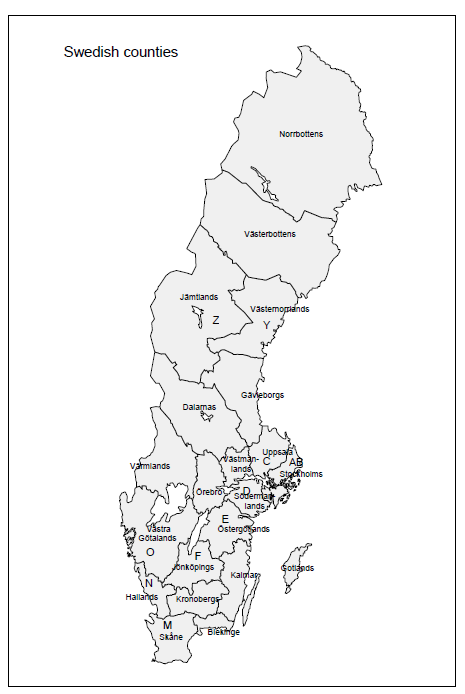


Additional file 1. Swedish counties with names and letter code where applicable.

Adapted from Statistics Sweden, [www.scb.se](http://www.scb.se)
