# Supplementary material for: Molecular characteristics of bovine norovirus and nebovirus in Swedish dairy herds
Source: Acta Vet Scand. 2025 Nov 13;67:46. doi: 10.1186/s13028-025-00830-9 (PMC12616915; doi:10.1186/s13028-025-00830-9)
Supplement: Supplementary file 2 — Supplementary Material 2 [file 13028_2025_830_MOESM2_ESM.docx]

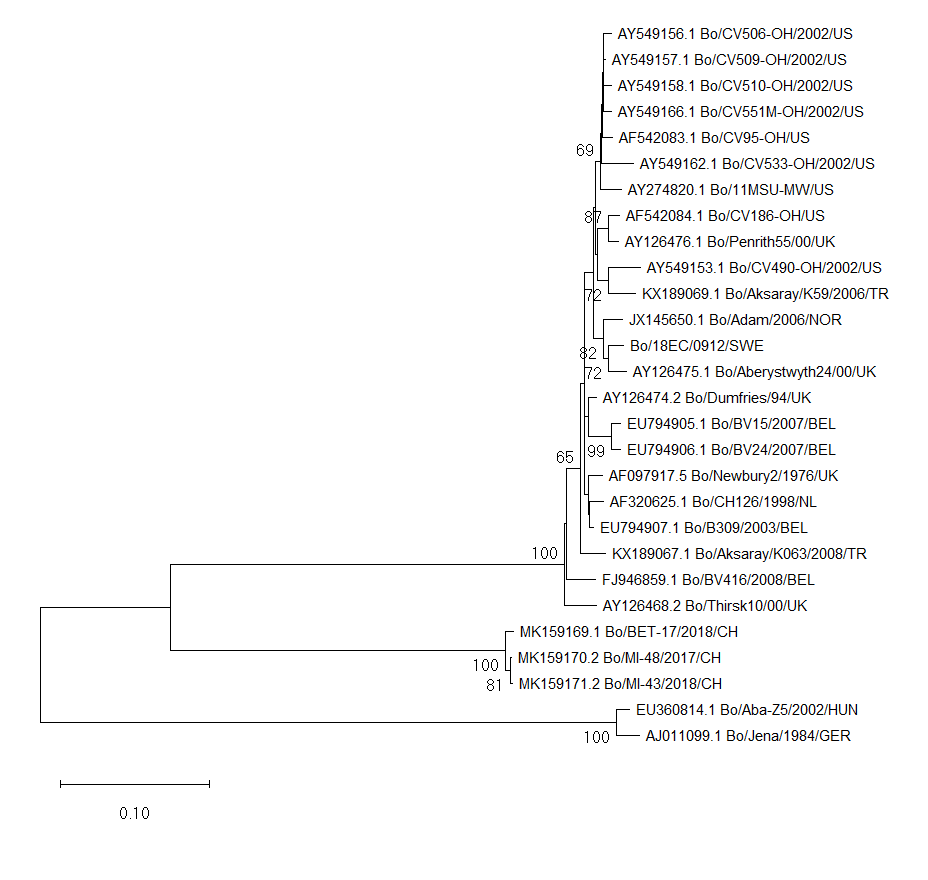


Additional file 2. Bovine norovirus VP1 GIII.1, 2, 4.

Neighbor-joining phylogenetic trees of the 522 aa complete VP1 (major capsid protein) sequence of a Swedish bovine norovirus and reference strains. Bootstrap values of nodes based on 1000 replicates, values >60 % shown. Analyses were performed in MEGA 11, showing GIII.1, GIII.2 and diverging Chinese VP1 sequences (GIII.4). The sequence designated SWE was detected in this study. Bar represents aa substitutions per site.
